# Individual-level socioeconomic status is associated with worse asthma morbidity in patients with asthma

**DOI:** 10.1186/1465-9921-10-125

**Published:** 2009-12-17

**Authors:** Simon L Bacon, Anne Bouchard, Eric B Loucks, Kim L Lavoie

**Affiliations:** 1 Montreal Behavioural Medicine Centre, Division of Chest Medicine, Research Center, Hôpital du Sacré-Cœur de Montréal - a University of Montréal affiliated hospital, 5400 Gouin West, Montréal, Québec, H4J 1C5, Canada; 2 Department of Exercise Science, Concordia University, 7141 Sherbrooke St West, Montreal, Quebec, H4B 1R6, Canada; 3 Montreal Behavioural Medicine Centre, Research Center, Montreal Heart Institute - a University of Montréal affiliated hospital, 5000 Belanger, Montreal, Quebec, H1T 1C8, Canada; 4 Department of Psychology, University of Quebec at Montreal (UQAM), PO Box 8888, Succursale Center-Ville, Montreal, Quebec, H3C 3P8, Canada; 5 Department of Community Health, Epidemiology Section, Center for Population Health & Clinical Epidemiology, Brown University, 121 South Main St, Providence, RI, USA

## Abstract

**Background:**

Low socioeconomic status (SES) has been linked to higher morbidity in patients with chronic diseases, but may be particularly relevant to asthma, as asthmatics of lower SES may have higher exposures to indoor (e.g., cockroaches, tobacco smoke) and outdoor (e.g., urban pollution) allergens, thus increasing risk for exacerbations.

**Methods:**

This study assessed associations between adult SES (measured according to educational level) and asthma morbidity, including asthma control; asthma-related emergency health service use; asthma self-efficacy, and asthma-related quality of life, in a Canadian cohort of 781 adult asthmatics. All patients underwent a sociodemographic and medical history interview and pulmonary function testing on the day of their asthma clinic visit, and completed a battery of questionnaires (Asthma Control Questionnaire, Asthma Quality of Life Questionnaire, and Asthma Self-Efficacy Scale). General Linear Models assessed associations between SES and each morbidity measure.

**Results:**

Lower SES was associated with worse asthma control (F = 11.63, p < .001), greater emergency health service use (F = 5.09, p = .024), and worse asthma self-efficacy (F = 12.04, p < .01), independent of covariates. Logistic regression analyses revealed that patients with <12 years of education were 55% more likely to report an asthma-related emergency health service visit in the last year (OR = 1.55, 95%CI = 1.05-2.27). Lower SES was not related to worse asthma-related quality of life.

**Conclusions:**

Results suggest that lower SES (measured according to education level), is associated with several indices of worse asthma morbidity, particularly worse asthma control, in adult asthmatics independent of disease severity. Results are consistent with previous studies linking lower SES to worse asthma in children, and add asthma to the list of chronic diseases affected by individual-level SES.

## Background

Asthma is a chronic disorder of the airways characterized by reversible and intermittent airway obstruction, airway inflammation, and hyper-reactivity of the airways in response to a variety of stimuli (e.g., dust, animal hair, smoke, and airborne pollutants). Despite important advances in diagnosis and treatment, asthma remains one of the most prevalent chronic respiratory disorders, affecting 7-10% of the world's population. Rather than decreasing, prevalence rates of asthma over the past three decades are actually rising in all age, sex, and racial groups in North America [1].

The global burden of asthma appears to be related to poor asthma control, which is associated with more frequent asthma symptomatology and bronchodilator use, worse pulmonary function, greater emergency health service utilization, and greater functional impairment (absenteeism, participation in social activities) [2,3]. In Canada, asthma remains poorly controlled in nearly 60% of patients, which places an excess burden on the health care system, and accounts for between 250-300 deaths per year [4,5]. Given that asthma can be well controlled for the vast majority of patients [2,3], identifying those patients who may be at greater risk for poorly controlled asthma represents an important goal for global asthma prevention.

Socioeconomic status (SES) has been linked to various health outcomes, with lower SES being associated with higher rates of morbidity and mortality from several chronic diseases, including cardiovascular disease, chronic obstructive pulmonary disease, and diabetes [6-8]. However, SES may be particularly relevant to asthma due to pathways by which it could adversely impact asthma outcomes. At the individual level (e.g., education attainment, income), asthmatics of lower SES may have higher exposures to indoor (e.g., cockroaches, tobacco smoke [9]) and outdoor (e.g., urban pollution [9]) allergens, and tend to use less inhaled corticosteroids [10], thus increasing risk for acute asthma exacerbations [9,11]. Though the SES-asthma link has been well established in children [12,13] and to some degree using area-level measures of SES (e.g., use of zipcodes or postal codes to define deprivation) in adults [14,15], less is known about associations between individual-level SES and asthma in adults.

The purpose of the present study was to assess associations between adult individual-level SES, measured according to education level, and several measures of asthma morbidity and health, including levels of asthma control, emergency health service use, asthma self-efficacy, and asthma-related quality of life in a Canadian cohort of asthmatics. It was hypothesized that SES would be significantly and negatively associated with these measures of asthma morbidity and health.

## Methods

### Study participants

A total of 781 consecutive adults with physician-diagnosed asthma (confirmed by chart evidence of a 20% fall in forced expiratory volume in 1 second (FEV_1_) after methacholine challenge and/or bronchodilator reversibility in FEV_1 _of ≥ 20% predicted [16]) were recruited from the outpatient asthma clinic of Hôpital du Sacré-Coeur de Montréal between June 2003-January 2007. Patients were eligible for the study if they were between the ages of 18 and 75 years and could communicate fluently in either French or English. Patients were excluded if they had occupational asthma, a co-morbid medical condition that significantly impacted health outcomes (e.g., cardiovascular disease, chronic obstructive pulmonary disease), evidence of severe psychopathology (e.g., schizophrenia), or cognitive deficits such that they could not give consent. A total of 1904 patients presented to the asthma clinic, of whom 1739 patients (91%) were screened for inclusion in the study (the remaining 165 patients had insufficient medical information with which to conduct pre-screening). A total of 885 patients were excluded (n = 358 due to existence of comorbid disease that conferred greater risk for morbidity than asthma, or the presence of severe psychopathology or substance abuse, n = 273 due to unconfirmed asthma or occupational asthma; n = 204 due to age criteria; and n = 50 due to language criteria), resulting in 854 eligible patients who were contacted to participate in the study. Only 53 patients declined to participate, which yielded a sample of 801 patients (94% participation rate). Twenty patients were excluded from analyses due to incomplete or missing data, yielding a final sample of 781 patients. This project was approved by the Ethics Committee of Hôpital du Sacré-Cœur de Montréal, and written consent was obtained from all participants.

### Study Design

This cross-sectional study was conducted as part of a larger study evaluating the prevalence and impact of psychiatric disorders among adult asthmatics [17]. Briefly, patients were screened to determine eligibility on the day of their regular asthma clinic. All patients underwent a sociodemographic interview (including questions about educational attainment), and a medical/asthma history interview (including assessments of height and weight for the calculation of body mass index, BMI) followed by a brief psychiatric interview (Primary Care Evaluation for Mental Disorders, PRIME-MD) that was administered by a trained, clinical research assistant. SES was measured according to educational level (total number of years completed), which is one of the most common measures of individual-level SES [18]. Educational attainment is frequently used as a measure of SES, because it is stable over time, unlike occupation and income that can fluctuate over the life course. Furthermore, participant response rates tend to be higher for educational attainment, unlike income which typically has lower response rates and consequently high response bias [18]. Asthma severity was classified based on Global Initiative for Asthma (GINA) guidelines [2] that classifies asthma severity into four categories (mild intermittent, mild persistent, moderate persistent, and severe persistent). To calculate asthma severity, all patients underwent standard spirometry to assess pulmonary function (FEV_1 _and forced vital capacity (FVC)). Patients completed a battery of questionnaires assessing asthma control (Asthma Control Questionnaire, ACQ), self-efficacy (Asthma Self-Efficacy Scale, ASES), and quality of life (Asthma Quality of Life Questionnaire, AQLQ). All self-reported clinical data, including medical/asthma history, asthma related hospital events, atopy status (based on skin prick testing [19]) and medication dosage, were verified by a clinical research assistant consulting the patient's medical chart.

### Questionnaire and Psychological Measures

#### Asthma Control Questionnaire (ACQ)

The ACQ [20] is a 7-item self-report questionnaire that assesses levels of asthma control in the last week according to standard criteria specified by international guidelines [2]. Items are rated on a 7-point scale, where 0 indicates good control and 6 indicates poor control, to yield a mean score out of 6. Patients are asked to report their symptoms, limitations in their daily activities, and bronchodilator use in the last week. FEV_1 _% predicted) was calculated from the pulmonary function test. The ACQ has demonstrated excellent measurement properties, has been validated in Canadian French, and scores of ≥ 0.8 indicate poorly controlled asthma [21]. For the current study, the internal consistency of the questionnaire was high (Cronbach's α = .84).

#### Asthma Self-Efficacy Scale (ASES)

The ASES [22] is an 80-item self-report questionnaire that assesses asthmatics' beliefs or confidence in their ability to successfully control or avoid an asthma attack in a variety of situations. The ASES is rated on a 5-point scale where 0 indicates "no confidence" and 4 indicates "very confident", to yield a final score out of 320 (with higher scores indicating better asthma self-efficacy). The ASES has shown to be a reliable and valid measure of asthma-specific self-efficacy and has been used extensively in previous studies [22,23]. For the current study, the internal consistency of the questionnaire was high (Cronbach's α = .98).

#### Asthma Quality of Life Questionnaire (AQLQ)

The AQLQ is a 32-item self-report questionnaire that assesses asthma-related quality of life across four life domains that may affected by asthma: symptoms, activity limitations, environmental stimuli, and emotional distress [24]. Items are rated on a 7-point scale, where 1 indicates very poor asthma-related quality of life and 7 indicates very good asthma-related quality of life, to yield a mean score out of 7. The AQLQ has demonstrated excellent measurement properties and has been validated in Canadian French [25]. For the current study, the internal consistency of the questionnaire was high (Cronbach's α = .96).

#### Primary Care Evaluation of Mental Disorders (PRIME-MD)

The PRIME-MD [26] assesses the prevalence (i.e., present or not) of mood (major and minor depression, dysthymia) and anxiety (panic disorders, generalized anxiety disorder, other anxiety disorder) using algorithms that are based on DSM-IV. It has been shown to be of comparable sensitivity, specificity and reliability as longer structured interviews, and takes approximately 10 to 20 minutes to administer and score [26].

### Analyses

Though main analyses were conducted using both continuous and dichotomous measures of education, sociodemographic, and medical/asthma history characteristics were presented as a function of low (<12 years of education) versus high (≥ 12 years of education) SES, i.e., the dichotomous measure. These cutoffs were chosen to reflect those who had (≥ 12) and had not (<12) completed high school. To assess the strength and direction of the association between SES as a continuous variable (i.e., years of education) and asthma morbidity measures (ACQ, ASES and AQLQ scores) a series of general linear models (GLM's) were conducted adjusting for age, sex, and asthma severity. In order to examine the robustness of our findings, an additional series of GLM's were conducted additionally adjusting for comorbid medical characteristics (current smoking, BMI, and having a mood and/or anxiety disorder [binary yes-no response]) that have been associated with worse asthma outcomes [17,27,28]. All covariates were determined a-priori. Two Poisson regression models was conducted to assess the relationship between years of education and emergency health service use [total number of emergency department visits and hospitalizations for asthma] in the past year, using the same covariates specified above. Theses models used a repeated statement in order to obtain robust standard errors for the Poisson regression coefficients. Finally, two logistic regressions were conducted to assess the impact of SES on the risk of emergency health service use in the last year (defined as a binary variable), using the same covariates specified above. All tests were two-sided and significance level was set at p < .05. Data analysis was performed using SAS v.9.1 (SAS Institute, Cary NC).

## Results

### Sample characteristics

The final sample of 781 patients included 467 (60%) women with a mean (SD) age of 48.5 (14.3) years. The mean (SD) duration of asthma for sample was 18.6 (15.2) years and 71% (n = 555) were atopic. The mean (SD) educational level was 12.9 (3.6) years (range 2-23 years) of schooling. Mean sample (SD) [range] for ACQ, ASES and AQLQ scores were 1.6 (1.1) [0.0-6.0], 222.3 (66.1) [13.3-320], and 5.1 (1.2) [1.5-7.0] respectively. A total of 184 (24%) of the sample reported a mean (SD) [range] of 2.1 (2.0) [1-15] emergency health service visits in the last year. Mean (SD) pulmonary function (% FEV1, %FVC, FEV1/FVC) for the sample was 78.9 (21.8), 89.5 (19.6), and 72.4 (14.4) respectively.

### Demographic and medical/asthma history characteristics

Demographic and medical/asthma history characteristics as a function of low (<12 years education) versus high (≥ 12 years education) SES are presented in table 1. Relative to patients with a higher SES, those with a lower SES were older and more likely to be unemployed. In addition, patients with a lower SES were more likely to engage in poor health behaviors, including being more likely to be current smokers, having a higher number of pack-years, and having a higher BMI.

**Table 1:** Demographic and medical/asthma characteristics presented as a function of high versus low SES

	Low (< 12 yrs education) n = 306		High (≥ 12 yrs education) n = 475			
				
	Mean or %	95% CI	Mean or %	95% CI	F	p
**Demographics**						
Age (yrs)*	52.3	50.8, 53.9	46.0	44.8, 47.3	37.42	< .001
Sex (% Male)	40	35, 46	40	36, 45	0.00	.997
Ethnicity (% Caucasian)	91	88, 94	92	90, 95	0.44	.518
Cohabitating (% yes)	68	62, 73	65	61, 70	0.47	.492
Employed (% yes)	49	43, 54	72	68, 76	45.73	< .001
**Medical characteristics**						
BMI (kg/m^2^)*	27.8	27.3, 28.4	26.6	26.1, 27.0	12.32	< .001
Current smoker (% yes)	13	9, 16	8	5, 10	5.75	.017
Pack-years†*	12.9	10.9, 14.9	6.6	5.0, 8.1	23.84	< .001
Psychiatric comorbidity (% yes)¶	32	27, 37	27	23, 32	2.02	.156
**Asthma characteristics**						
FEV1 (% predicted)*	77.3	74.6, 79.9	79.9	77.8, 82.0	2.32	.129
FVC (% predicted)*	87.8	85.3, 90.2	90.5	88.6, 92.4	3.03	.082
FEV1/FVC*	71.4	69.6, 73.2	72.9	71.6, 74.3	1.78	.183
Asthma severity (% moderate or severe)	92	88, 96	84	80, 87	10.94	.001
Asthma duration (yrs)*	18.2	16.5, 19.9	18.9	17.5, 20.3	0.45	.502
Atopic (% yes)	62	57, 67	77	73, 81	23.01	< .001
Bronchodilator use (# times in last week)*	9.6	7.9, 11.3	7.0	5.6, 8.3	5.77	.017

FEV1 = forced expiratory volume in 1 second; FVC = forced vital capacity; BMI = body mass index;

* These values are reported as means

† pack-years = average number of packs (20 cigarettes/pack) smoked per day X number of years smoked;

¶ Mood and-or anxiety disorder

With regards to asthma, patients with a lower SES were less likely to be diagnosed with atopy but were more likely to have moderate or severe (relative to intermittent or mild) asthma, and took their bronchodilator significantly more often than higher SES patients.

### Association between SES, asthma morbidity and health

Associations between SES and asthma morbidity variables are presented in Table 2. GLM analyses revealed that that lower SES was negatively associated with higher ACQ scores (i.e., worse asthma control) and lower ASES scores, independent of age, sex, and asthma severity. When additional covariates (current smoking, BMI, and having a mood and/or anxiety disorder) were added to the model, lower SES remained significantly associated with higher ACQ scores and lower ASES scores. In addition, there was an approximate 30% reduction in the β after adjusting for covariates, suggesting these variables accounted for some but not all of the association strength There were no associations between SES and AQLQ scores. Poisson regression revealed that lower SES was associated with greater emergency health service use, independent of age, sex, asthma severity (estimate = -0.07, SE = 0.02, 95%CI = -0.10- -0.03), and all additional covariates (estimate = -0.05, SE = 0.02, 95%CI = -0.09- -0.01), with a minimal change in the estimate. Logistic regression analyses revealed that patients with < 12 years of education were 55% more likely to report being hospitalized or having an emergency department visit in the last year (OR = 1.55, 95%CI = 1.05-2.27), independent of age, sex, and asthma severity. When factors expected to mediate the association between education and asthma severity (i.e. current smoking, BMI, and having a mood and/or anxiety disorder) were added to the model, the magnitude of the effect was slightly reduced and became no longer statistically significant (OR = 1.46, 95%CI = 0.98-2.17).

**Table 2:** Association between educational attainment and asthma morbidity variables (GLM)

	model adjustment					
	
	age, sex, asthma severity			age, sex, asthma severity, BMI, smoking, and psychiatric disorder		
		
	β (SE)	F	p	β (SE)	F	p
ACQ	-0.041 (0.011)	14.12	< .001	-0.027 (0.011)	5.82	.016
ASES	2.797 (0.787)	12.65	< .001	2.110 (0.795)	7.04	.008
AQLQ	0.021 (0.014)	2.34	.127	0.004 (0.014)	0.08	.783

ACQ = Asthma Control Questionnaire; ASES = Asthma Self-Efficacy Scale; AQLQ = Asthma Quality of Life Questionnaire.

## Discussion

The present study assessed associations between individual-level SES (measured according to educational attainment) and multiple measures of asthma morbidity in a Canadian cohort of adult asthmatics. Results showed that patients with lower SES had worse asthma control, worse asthma self-efficacy, and greater emergency health service use relative to patients with higher SES, independent of age, sex, asthma severity, current smoking, BMI, and having a mood and/or anxiety disorder. We also found that patients with less than 12 years of education were 55% more likely to report any emergency health service use, compared to those with 12 or greater years of education, when controlling for age, sex, and severity. When the additional covariates were included in the model, this relationship was no longer statistically significant. However, though statistical significance was lost, there was a minimal change in the point estimate, suggesting that mediation was unlikely. Furthermore, the Poisson regression models indicated that the relationship between education and emergency healthcare usage may be graded and have a dose-response association, even with the inclusion of all covariates.

These findings are consistent with previous studies finding significant associations between lower childhood SES and worse asthma morbidity, including increased prevalence of asthma and severe asthma [12,13], and increased risk of emergency department visits and hospitalizations for asthma [29,30]. These findings are also in line with previous studies linking lower SES (assessed using area-level and individual-level measures) to worse asthma morbidity in adults, including increased prevalence of asthma [31], greater asthma symptomatology [32], and increased asthma related hospitalisations [33]. However, this study is, to our knowledge, the first to assess the impact of individual-level SES on multiple measures of asthma morbidity in such a large Canadian cohort of adult asthmatics. Although Lynd et al. [34] examined the link between both individual and area-level measures of SES and asthma in a Canadian sample, their sample size was modest (n = 202), and their analyses focused on links between SES and short-acting bronchodilator use as a proxy measure of asthma control. Their findings are still consistent with those of our study, though we were able to extend their findings by showing that asthmatics of lower SES have worse asthma control according to the ACQ and emergency health service use.

It is noteworthy that patients with lower SES were more likely to exhibit poor health behaviors that may exacerbate asthma, including higher rates of current smoking, total pack-years, and BMI. This is consistent with previous studies linking higher rates of smoking, obesity, reduced consumption of fruits and vegetables, and higher consumption of saturated fats in low SES individuals compared to high SES individuals [35-37]. The higher prevalence of poor health behaviors among socially disadvantaged adults with asthma may partially explain why these patients were more poorly controlled. However, the fact we found lower SES to be related to worse asthma control after adjustment for BMI and smoking suggests these were not the only potential mechanisms linking lower SES to poor control in this study. For example, and as detailed above, nutrition may also play a role. It must also be noted that our assessments of smoking and BMI may be imperfect (e.g., central adiposity may be more important than total body composition). Though the current study was not designed to assess the potential mechanisms linking lower SES to increased asthma morbidity, they can be found in previous studies. For example, lower SES was associated with lower use of inhaled corticosteroids [10] and lower corticosteroid adherence [38], though not all studies have reported this [39]. The current study did not collect data on medication adherence, but the results were independent of asthma severity, which is primarily derived from the prescribed dosage of inhaled corticosteroids. Furthermore, a previous study has shown that SES was related to ACQ scores independent of corticosteroid use [40]. There is also evidence that the underlying physiological processes seen in asthma are influenced by SES, where heightened inflammatory responses to similar doses of antigen challenge have been shown in patients with low versus high SES [41,42], which may be a consequence of low SES individuals overexpressing genes regulating their inflammatory processes [43]. However, it should be noted that these findings are drawn from data in children and needs to be replicated in adult samples.

One additional finding that warrants discussion is that asthmatics of lower SES were less likely to be atopic (i.e., have allergic asthma) than asthmatics of higher SES. Although this was not the primary aim of the analyses, this finding is consistent with several studies linking lower SES to lower incidence of allergic asthma [31,32,44,45]. Although controversial, it has been suggested that this relationship may be due to the "hygiene hypothesis," which proposes that the development of atopic asthma and allergy may be prevented via prenatal and-or early childhood exposure to immune system stimulants (e.g., bacteria, viruses and endotoxins) that shift T-helper type 2 cell (Th2) dominance to T-helper type 1 cell (Th1) dominance [46,47]. This shift in cytokine balance is thought to contribute to allergic asthma and allergy, and may be induced by a lack of early exposures to microbial environments [46], which are typical in lower SES settings (e.g., poor housing conditions that may be overcrowded, infested with cockroaches and dust mites, and poorly insulated, leading to greater exposure to infections, allergens, and mould). Our finding of less atopic asthma in patients of lower SES may therefore lend support for the "hygiene hypothesis." However, given the fact that this is a secondary finding, and the controversies surrounding the "hygiene hypothesis," further investigation is clearly needed.

Surprisingly, we did not observe any significant association between SES and asthma-related quality of life, which was contrary to our expectations and to previous findings [14,48]. Both lower area-level SES [14] and composite individual-level SES [48] have been associated with worse general and asthma-specific quality of life. The reasons for these inconsistencies are not clear. However, they may be related to issues associated with the nature of the populations assessed and to study design. For example, Blanc et al. [14] recruited patients from multiple clinics via physician referral, as well as using random-digit telephone recruitment; whereas we recruited consecutive patients from a single tertiary-care clinic where asthma is generally more severe and thus may reduce variability in quality of life measures. The Apter et al. [48] study found that the relationship between SES and quality of life was highly confounded by race/ethnicity, with non-Caucasians having lower SES and poorer quality of life. While the Apter et al. study consisted of nearly 60% of non-Caucasians, the current study has less than 10% non-Caucasions, suggesting that the results reported by Apter et al. may have been driven by race/ethnicity rather than SES [49]. In addition, the significant association between SES and worse asthma-specific quality of life in Blanc et al.'s study was observed using a different measure of SES (i.e., area-level), and a different quality of life scale (i.e., Marks Asthma Quality of Life Questionnaire) than those used in the present study. As such, the disparate findings between these two studies may be attributable to the specific choice of measures. Further replication studies are needed to shed more light on the association between SES and asthma-related quality of life in adult samples.

The results of this study need be interpreted in consideration of some methodological limitations. First, patients were recruited from the asthma clinic of a single tertiary-care urban hospital, so results may not generalize to rural centers or community samples. Second, we relied upon education level as our measure of individual-level SES, when it may have been more informative to use a composite measure (e.g., education level, income, and-or occupation), or to triangulate analyses using occupation and income as separate measures of SES. Unfortunately, the only additional variable we collected was on employment status (yes-no). In addition, it should be noted that that education is the most common measure of individual-level SES and is stable over time, unlike occupation and income, that can fluctuate over the life course. Furthermore, participant response rates tend to be higher for educational attainment, unlike income which typically has lower response rates and consequently high response bias [18]. Third, the study was cross-sectional so reverse causality may be possible, though unlikely, and education and asthma morbidity may be linked in a non-causal fashion. As such, further longitudinal studies are needed to confirm the temporal sequence of the results in the current study. Finally, our study was limited by the fact that we were not able to assess other environmental variables that are associated with SES that may have partially accounted for our findings such as actual exposure levels to allergens, irritants, and pollutants, and living conditions (i.e., overcrowding) which may have increased the risk of respiratory infections that confer risk for worse asthma morbidity [32]. Despite these limitations, the results of the present study complement and strengthen previous reports by including a large cohort of adult asthmatics with objectively confirmed physician-diagnosed asthma and atopy, and the measurement of a range of asthma morbidity and health measures that included self-reported symptoms and objectively measured emergency health service utilization that was verified by chart review. Due to the range and depth of our assessments, we were also able to control for a number of potential confounders, including smoking status, BMI, psychiatric comorbidity, and asthma severity, which attests to the robustness of the findings.

## Conclusions

In summary, this study found evidence for an association between education level (which is indicative of SES) and asthma morbidity and health in a large tertiary-care sample of Canadian adults with asthma, with lower education levels being related to worse levels of asthma control and asthma self-efficacy, and higher rates of emergency health care use for asthma in the past year. As this study was not designed to examine the mechanisms linking SES to asthma morbidity, future studies should examine the pathways by which SES influences asthma morbidity among adults and the extent to which they may differ from the pathways proposed in children. In addition, while directly intervening on SES is difficult, once the mechanisms of the SES-asthma relationship have been identified interventions need to be developed to improve asthma outcomes in low SES patients [50].

## Competing interests

The authors declare that they have no competing interests.

## Authors' contributions

SLB co-wrote the manuscript, conducted all statistical data analyses, and obtained funding for the study. AB collected primary data and helped develop the conceptual idea. EBL helped develop the conceptual framework and provided critical feedback on manuscript drafts. KLL conceived of the study, participated in its design and coordination, obtained funding for the study, and co-wrote the manuscript. All authors read and approved the final manuscript.
